# Fully Ab-Initio Determination of the Thermoelectric Properties of Half-Heusler NiTiSn: Crucial Role of Interstitial Ni Defects

**DOI:** 10.3390/ma11060868

**Published:** 2018-05-23

**Authors:** Alexandre Berche, Philippe Jund

**Affiliations:** Institut Charles Gerhardt Montpellier (ICGM), Centre National de la Recherche Scientifique (CNRS), Université de Montpellier, Ecole Nationale Supérieure de Chimie de Montpellier, UMR 5253, Montpellier, France; alexandre.berche@gmail.com

**Keywords:** thermoelectric materials, half-Heusler phase, point defects

## Abstract

For thermoelectric applications, *ab initio* methods generally fail to predict the transport properties of the materials because of their inability to predict properly the carrier concentrations that control the electronic properties. In this work, a methodology to fill in this gap is applied on the NiTiSn half Heusler phase. For that, we show that the main defects act as donor of electrons and are responsible of the electronic properties of the material. Indeed, the presence of Ni_i_ interstitial defects explains the experimental valence band spectrum and its associated band gap reported in the literature. Moreover, combining the DOS of the solid solutions with the determination of the energy of formation of charged defects, we show that Ni_i_ defects are also responsible of the measured carrier concentration in experimentally supposed “pure” NiTiSn compounds. Subsequently the thermoelectric properties of NiTiSn can be calculated using a fully *ab initio* description and an overall correct agreement with experiments is obtained. This methodology can be extended to predict the result of extrinsic doping and thus to select the most efficient dopant for specific thermoelectric applications.

## 1. Introduction

In order to fight against global warming, new energy sources have to be developed. In that spirit, thermoelectricity can directly convert waste heat into electricity using the Seebeck effect. The efficiency of thermoelectric modules is directly linked to the figure-of-merit (*ZT*) of the semiconductors (respectively n and p) composing the module. This figure-of-merit is given by *ZT = S^2^σT/κ* where *T* is the temperature, *S* the Seebeck coefficient, *σ* and *κ* the electrical and thermal conductivity respectively. One important aspect for the sustainability of an energy source is to use non toxic, recyclable and abundant elements. Therefore, materials such as half Heuslers are good candidates. Due to its valence electrons counting (VEC) the ternary phase NiTiSn and its derivates is a potential candidate as it can be doped in order to improve its *ZT* up to 1.5 at 650–700 K [1].

The research of the best doping element is usually done by experimental tests. However, from an experimental point of view, synthesis and characterization may be sensitive. This is especially the case for NiTiSn. Indeed, this phase is generally obtained by liquid routes which lead to multiphased alloys even after long annealing times. Such secondary phases are known to decrease the *ZT* of the material [2]. This is why alternative routes for the synthesis, such as ball milling [3] may be useful to obtain the pure phase. In addition, NiTiSn is also known to be highly sensitive to oxidation [4] which can also have high effects on its thermoelectric properties. In parallel of experimental investigations, *ab initio* methods may also provide interesting elements in the choice of the doping elements. However, this interest is limited since, to our knowledge, the *ZT* of a compound has never been obtained from a fully *ab initio* point of view. The present study aims to fill this lack. To calculate the *ZT* fully *ab initio*, we need to combine electronic properties calculations (Seebeck coefficient *S*, electrical conductivity *σ* and the electronic part of the thermal conductivity *κ_e_*) with phonon calculations (lattice part of the thermal conductivity *κ_l_*). This last contribution has already been calculated several times [5,6,7] for NiTiSn and will not be investigated again in the present paper. The electronic transport properties will be obtained by solving the Boltzmann’s equations within the constant relaxation time approximation. The main limitation in this theory consists in the usual use of experimental carrier concentrations (*N*). To avoid this, we will assume that the thermoelectric properties of the non-doped material are due to electrons (or holes) provided by the main intrinsic defects. It is then possible to estimate the value of *N* by calculating the energy of formation of charged defects as previously reported [8]. In NiTiSn, the most probable defect consists in interstitial Nickel atoms (Ni_i_) present on the *4d* Wyckoff positions (3/4; 3/4; 3/4) of the half-Heusler phase (space group $F\bar{4}3m$ , prototype MgAgAs) as predicted by Density Functional Theory (DFT) calculations [4,9,10] as well as experimental studies [11,12,13]. This paper will focus on the influence of these Ni_i_ defects on the electronic properties (Density of States (DOS) and bandgap) and on the thermoelectric properties.

## 2. Methods

The DFT calculations are performed using the Vienna *ab initio* simulation package (VASP) [14,15] and the projector augmented waves (PAW) technique [16,17] within the generalized gradient approximation (GGA). The Perdew–Burke–Ernzerhof parameterization (PBE) is applied [18,19].

Standard versions of the PAW potentials for Ni, Sn and Ti are used. The pseudo-potential names are respectively Ni, Sn_d, Ti_sv. Ten electronic states are included in the valence shell for Ni (3d^8^4s^2^), twelve for Ti (3s^2^3p^6^3d^2^4s^2^), whereas fourteen valence electrons are considered for Sn (4d^10^5s^2^5p^2^).

The calculations are performed using the “accurate” precision setting in the VASP input file (energy convergence: 10^−5^ eV/cell; force convergence: 10^−4^ eV/Å). For point-defects calculations, a 2 × 2 × 2 supercell of the cubic conventional cell is considered leading to a total of 96 atoms. The integrations in the Brillouin zone are performed using Monkhorst–Pack k-point meshes. The reciprocal space mesh is set to 7 × 7 × 7 leading to 20 k-points in the irreducible part of the Brillouin zone. The cutoff energy is set to 500 eV for the whole study. Since the NiTiSn phase is non-magnetic (even with defects such as Ni_i_), spin-polarization is not taken into consideration.

The energy of formation (*Δ_f_E(phase)*) of a *A_x_B_y_C_z_* phase is calculated using Equation (1) where *E(phase)*, *E(A)*, *E(B)* and *E(C)* is the DFT-calculated energy of the phases *A_x_B_y_C_z_*, *A*, *B* and *C* respectively in their standard crystallographic structure and *N_cell_* is the number of atoms in the *A_x_B_y_C_z_* cell. In this equation, the energies are in eV/cell for *A_x_B_y_C_z_* and in eV/atom for *A*, *B* and *C*.

(1) $$\Delta_{f}E\left(A_{x}B_{y}C_{z}\right)=\frac{E\left(A_{x}B_{y}C_{z}\right)}{N_{cell}}-\frac{xE\left(A\right)+yE\left(B\right)+zE\left(C\right)}{x+y+z},$$

The electronic transport properties are analyzed by using the BoltzTraP code (version 1.2.5: Madsen+Singh-www.imc.tuwien.ac.at) under the constant relaxation time approximation [20]. More details will be given in Section 5.

## 3. Influence of Interstitial Ni Defects on the Electronic Structure

**Figure 1:**
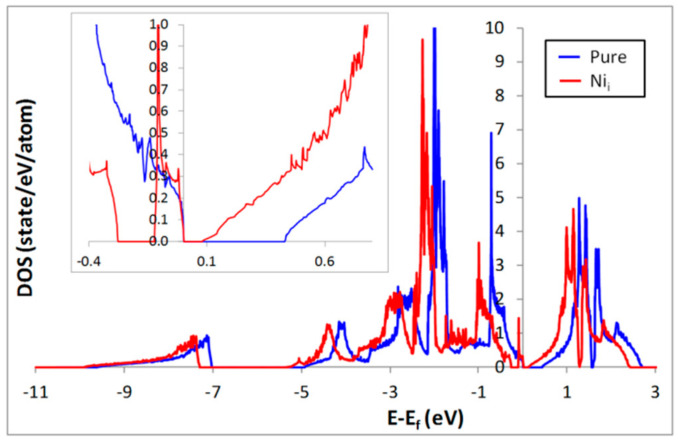
DOS of NiTiSn calculated in GGA for the pure compound and for the compound containing 1% of interstitial nickel atoms (Ni_i_).

To estimate the electronic transport properties of a compound, we need a correct description of its electronic structure as well as of its gap. For the pure NiTiSn phase, the calculated DOS is given in Figure 1. The gap is estimated at 0.43 eV in agreement with previous GGA calculations (0.45 eV [21], 0.416 eV [9]). However, even if generally the GGA is supposed to underestimate the value of the gap, in this case it has been measured at 0.12 eV [22]. If this difference between the DFT result and the experimental value comes from the functional as is often the case [23], then a hybrid functional such as HSE06 [24] may give a better description of the bandgap. Calculations with HSE06 [25] predict a gap of 0.61 eV for the pure NiTiSn phase. So GGA underestimates the bandgap in comparison to HSE06 but the calculation method does not give an explanation for the difference between the calculated and experimental values.

In experimental samples defects such as Ni_i_ (the most favorable defects) may be present [12], so it is reasonable to compare the calculated gap for the system containing Ni_i_ defects (Ni_1+x_TiSn) to the experimental one. For that, we have considered a single interstitial Ni atom in a 2 × 2 × 2 supercell containing 96 atoms leading to a defect concentration of around 1%. In Figure 1 the DOS of this system is shown and it can be noticed that Ni_i_ generates additional states inside the band gap, in agreement with previous calculations [9]. Therefore the gap is calculated at 0.08 eV in agreement with the previous results of Colinet et al. [9] (approximately 0.12 eV). Thus the presence of the interstitial Ni atoms can explain the experimental gap of 0.12 eV even if our GGA calculations slightly underestimate the gap (as expected). These calculations are consistent with the presence of the additional states located in the energy gap region experimentally reported by Miyamoto et al. [26]. A Similar observation has also been reported for the NiZrSn half-Heusler phase [27].

## 4. Determination of the Energy of Formation of Defects

To calculate the electronic transport properties of a phase, we have to combine the electronic structure with the carrier concentration *N* in the compound. For that, we assume that the intrinsic defects are responsible of the electronic properties of the non doped compound. Indeed, intrinsic defects will act as carrier donors or acceptors. The carrier concentration can be estimated by using Equation (2) where *V* is the volume of the supercell, *n_h_* and *n_e_* are the number of holes and electrons respectively in the cell, *q* the charge (in number of electrons) and *n_D_* is the number of defects D per formula unit and *μ_e_* the chemical potential of the electrons. This last term is defined by Equation (3) where *N_site_* is the number of defect sites per formula unit of the crystal, *N_sym_* is the number of symmetrically equivalent ways of introducing the defect on one defect site (*N_sym_* = 1 for defects involving one atom such as vacancies or atomic substitutions), *Δ_def_E_charged_* the energy of formation of the charged defect, *k_B_* the Boltzmann constant and *T* the temperature.

(2) $$N\left(T\right)=\frac{1}{V}\left(n_{h}\left(T\right)-n_{e}\left(T\right)\right)=-\frac{1}{V}\sum_{D}qn_{D}\left(T\right),$$

(3) $$n_{D}\left(T\right)=N_{site}N_{sym}exp\left(-\frac{\Delta_{def}E_{charged}}{k_{B}T}\right).$$

To obtain the *ab initio* calculated carrier concentration, one needs to define *μ_e_* and *Δ_def_E_charged_*. Several methods have been suggested in the literature to calculate the energy of formation of a charged defect (*Δ_def_E_charged_*). These methods will be described here for a non-charged defect in Section 4.1; the effect of the charge will be considered in Section 4.2.

### 4.1. Case of a Non-Charged Defect

The simplest method (*Δ_def_E_s_*) consists in calculating the difference of the energies of formation of Ni_1+x_TiSn (*Δ_f_E(def)*) and the pure NiTiSn (*Δ_f_E(pure)*). This difference is divided (Equation (4)) by the concentration of defects in the cell *x_def_* as it has been previously described [9,28]. In this equation, *Δ_f_E(def)* and *Δ_f_E(pure)* are calculated at −0.5386 eV/at and −0.5469 eV/at respectively using Equation (1).

(4) $$\Delta_{def}E_{s}=\frac{\Delta_{f}E\left(def\right)-\Delta_{f}E\left(pure\right)}{x_{def}}.$$

This method is simple, but it does not take into consideration the phases which are in equilibrium with the compound containing defects [4,29]. To correct this, two methodologies can be used. In the first one, one assumes that adding a defect in the structure does not change the global composition of the sample. In these conditions, at infinite dilution, the influence of the chemical potentials of the pure elements and of the other phases is taken into consideration. This formalism has been described by Zhang and Northrup [8]. With this method, the energy of formation of a non-charged defect (*Δ_def_E_μ_*) is given by Equation (5) where *i* is the element involved in the defect; *E_def_* and *E_perfect_* are the total energy (eV/cell) of a non-charged defect in the supercell and the perfect supercell respectively; *ΔN_i_* is the difference in the number of atoms induced by the defect; *E_i_* is the energy (eV/at) of element i in its standard state; *Δμ_i_* is the chemical potential of element *i* (eV/at).

(5) $$\Delta_{def}E_{\mu }=E_{def}-E_{perfect}-\sum_{i}\Delta N_{i}E_{i}-\sum_{i}\Delta N_{i}\Delta \mu_{i}.$$

**Figure 2:**
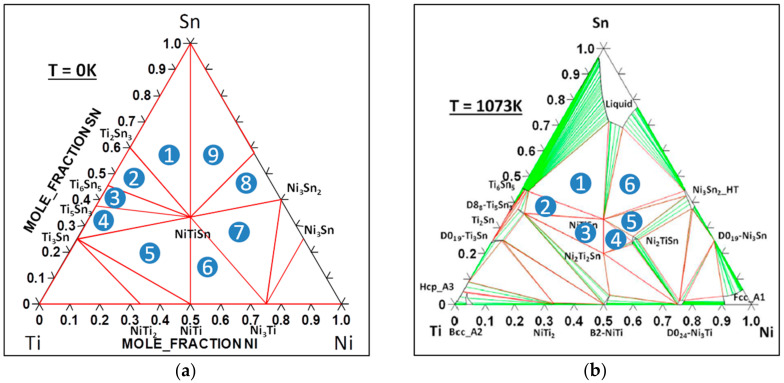
Isothermal phase diagram of the Ni-Sn-Ti system: (**a**) At 0 K, calculated in GGA; (**b**) at 1073 K plotted using the Calphad method [30].

To use this formalism, it is necessary to calculate the chemical potential of each element in the phase. However, since the chemical potential of an element in a phase changes with the composition, one needs to estimate the chemical potential from limiting cases defined in 2-phased regions (for a binary system) or 3-phased regions (for a ternary system). As a consequence, the calculated *Δ_def_E_μ_* depends on the phase regions considered. This means that in order to obtain the correct energy of formation of a defect, one needs a precise knowledge of the phase diagram and of the energy of formation of each binary and ternary phase in equilibrium with the compound. In the case of NiTiSn, the phase diagram has been described and the energy of formation of the phases has been calculated and measured [4,11,30]. To be consistent with our previous work, the energies calculated within the GGA [30] will be considered here. However, it has been shown that the phase equilibrium estimated at 0 K from GGA calculations (Figure 2a) significantly differs from the experimental one (see Figure 2b in which the considered phase diagram is the assessed one using the Calphad method [30]). The differences mainly arise from an overestimation of the energy of formation of NiTiSn within the GGA which can be corrected using a GGA + U description for example [4]. Since the choice of the phase diagram is important for the calculated energy of formation (and then for the carrier concentration and the thermoelectric properties) we will consider both versions in this study.

For each of the considered phase diagrams, *Δ_def_E_μ_* for Ni_i_ is calculated in all the 3-phased regions (9 for the GGA phase diagram and 6 for the Calphad one) in equilibrium with NiTiSn (Table 1). From these calculations it becomes obvious that *Δ_def_E_μ_* depends on the phase region considered since the value changes by a factor of 2.4 and 2.9 respectively for the GGA and Calphad phase diagram. It can be noticed that the energy of formation of the non-charged defect (going from 0.405 to 1.172 eV) is similar to the one previously calculated by Stern et al. [30] going from 0.52 to 1.03 eV according to the phase region considered.

To avoid the limitations of the two previous formalisms, it is possible to modify Equation (4) by replacing the *Δ_f_E(pure)* by the *Δ_f_E(multi)* as suggested in our previous works [4,29]. In this third formalism, we assume that the defect will change the global composition of the alloy. As a consequence, Equation (4) becomes Equation (6). With such a formalism, the phases in equilibrium with our compound will be taken into consideration through the energy of formation of the multiphased region. In Equation (6), *Δ_f_E(multi)* is the energy of formation of the multiphased region corresponding to the EXACT composition of the cell with the defect Ni_1+x_TiSn (no infinite dilution is assumed). This term is described in Equation (7) where *φ* designates the phases involved in the multiphased region, *x_φ_* the fraction of phase *φ* in the multiphased region and *Δ_f_E(φ)* the energy of formation of each phase calculated using Equation (1).

(6) $$\Delta_{def}E_{multi}=\frac{\Delta_{f}E\left(defect\right)-\Delta_{f}E\left(multi\right)}{x_{defect}},$$

(7) $$\Delta_{f}E\left(multi\right)=\sum_{\varphi }x_{\varphi }\Delta_{f}E\left(\varphi \right).$$

It is worth noting that the single energy calculation with *Δ_def_E_multi_* gives the exact same result as the lowest value obtained for *Δ_def_E_μ_* (requiring 6 or 9 calculations).

In addition, the energy of formation of defect *Δ_def_E_s_* overestimates *Δ_def_E_multi_* by a factor 1.7 to 2. In the following parts of the paper, the *Δ_def_E_multi_* formalism will be considered.

### 4.2. Case of a Charged Defect

In the case of a charged defect, the total energy of the cell has been recalculated with the modified number of electrons. This can be easily done in a software such as VASP. However, the main disadvantage of this method is that it is not possible to localize the charge on the defect. The total energy of the supercell with the defect (*E_def_*) has been corrected ( $E_{der}^{corrected}$ ) by taking into consideration the charge effect. In this work, two additional correction terms are taken into account. The first one is the electrostatic interaction generated by repeated defects due to the periodic boundary conditions mimicking an infinite system (*E_Madelung_*); the second one is the potential alignment (*ΔV*) to refer the charged supercell to the pure supercell (as defined by Taylor and Bruneval [31]). The corrected energy of the charged supercell is given by Equation (8) where *q* is the number of charges (in electron), *ε_VBM_* the maximum of the valence band (in eV) of the pure cell, *μ_e_* is the chemical potential of the electrons (in eV).

(8) $$E_{def}^{corrected}=E_{def}+E_{Madelung}+q\left(\varepsilon_{VBM}+\Delta V+\mu_{e}\right).$$

The two additional terms are given by Equations (9) and (10) respectively where *α* is the Madelung’s constant, *L* is the distance between defects (cell parameter of the supercell in the case of NiTiSn), *ε* is the relative permittivity of the pure phase, $\nu_{KS}^{bulk}$ and $\nu_{KS}^{defect}$ are the Kohn–Sham potentials of the pure and charged cell respectively.

(9) $$E_{Madelung}=\frac{\alpha }{2L\varepsilon }q^{2},$$

(10) $$\Delta V=\nu_{KS}^{bulk}-\nu_{KS}^{defect}.$$

In this work, the Madelung’s constant for NiTiSn is taken at 2.51939, value tabulated for the CaF_2_ structure which is similar to the half-Heusler structure. The relative permittivity has been calculated by the density functional perturbation theory implemented in VASP. The calculated value is 25.96, a value higher than the ones calculated within the GGA (22.51 [32]) or within the Local Density Approximation (LDA) (19.88 [32]) using a similar methodology. However, our value is in better agreement with the 36.5 measured experimentally [33]. In the following the value calculated in this work will be used.

**Figure 3:**
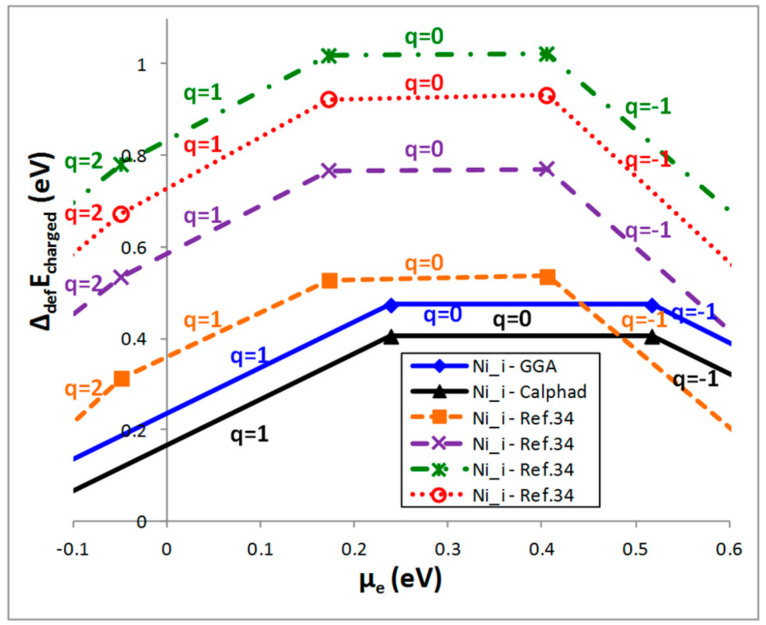
Evolution of *Δ_def_E_charged_* for Ni_i_ defects with the chemical potential of the electrons for different values of the charge q calculated for one defect in a 2 × 2 × 2 supercell taking into consideration the phase diagram calculated in GGA or assessed with the Calphad method.

The values of $E_{def}^{corrected}$ are then calculated for different values of the charge. This energy is used for calculating the energy of formation of Ni_i_ defects with Equation (6). The result is plotted as a function of *μ_e_* which generally varies in the vicinity of the band edges. For each value of *q* (ranging from −2 to 2 in this study), *Δ_def_E_multi_* has a linear variation with *μ_e_* with a slope *q*. In Figure 3 we have only plotted the most stable cases (represented by sections of these lines). For comparison, previous values published by Stern et al. [34] (using the *Δ_def_E_μ_* formalism) have been added. Their different sets of data correspond to different compositional regions (Ni rich, Ni poor, etc.). If the absolute value of *Δ_def_E_charged_* at *μ_e_* = 0 highly differs from the one calculated by Stern et al. [34], all the results show that the most stable charged Ni_i_ defect is obtained for *q* = +1 which is typical of an n-type semiconducting material and consistent with all the experimental measurements.

## 5. Ab-Initio Calculation of the Thermoelectric Properties

### 5.1. Calculation of the Carrier Concentration N

It is now possible to calculate the carrier concentration generated by Ni_i_ defects in NiTiSn using Equations (2) and (3). Before that, one needs to estimate the chemical potential of the electrons *μ_e_*. This is achieved by solving Equation (2) on the basis of the DOS of NiTiSn. Indeed, the number of holes *n_h_(T)* and the number of electrons *n_e_(T)* can be directly deduced from the integration of the DOS as given in Equations (11) and (12) respectively, where *n(ε)* is the total DOS considered, *f(ԑ,T)* the Fermi-Dirac distribution, *T* the temperature and *ε_VBM_* and *ε_CBM_* the energy of the valence band maximum and of the conduction band minimum respectively. In this work, *μ_e_* is referred to *ε_VBM_*.

(11) $$n_{h}\left(T\right)=\int_{-\infty }^{\varepsilon_{VBM}}n\left(\varepsilon \right)\left(1-f\left(\varepsilon ,T\right)\right)d\varepsilon ,$$

(12) $$n_{e}\left(T\right)=\int_{\varepsilon_{VBM}}^{\infty }n\left(\varepsilon \right)f\left(\varepsilon ,T\right)d\varepsilon .$$

**Figure 4:**
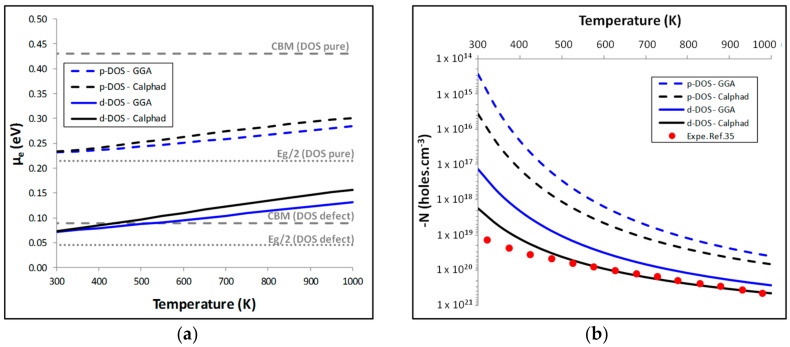
Evolution with temperature of the calculated: (**a**) *μ_e_*; (**b**) *N* induced in NiTiSn by Ni_i_ defects.

It becomes then obvious that the choice of the DOS will have an influence on the calculated carrier concentration *N*. As discussed in Section 3, the most accurate DOS is the one with the defect since it allows to give the best description of the experimental DOS and of its associated band gap. However, we have decided to do the calculation of *N* with the pure DOS (p-DOS) and with the DOS with the defect (d-DOS) to estimate the influence of this choice on *N*. The value of *μ_e_* is then adjusted at each temperature to fulfill Equation (2) using the energy of formation of charged defects calculated using the GGA phase diagram or the one assessed by the Calphad method. The results (Figure 4a) show that at room temperature the chemical potential of the electrons is located between the mid-gap (*E_g_*/2) and the conduction band maximum (CBM). As temperature increases, *μ_e_* increases too and even crosses the CBM if the d-DOS is used to estimate *μ_e_*. Such a behavior is typical of n-type semiconductors.

The carrier concentration *N* is finally calculated using Equations (2) and (3). The results are compared to experimental data estimated in the literature from Hall measurements [35] in Figure 4b. The carrier concentration, calculated with the d-DOS and the phase relationship obtained with the Calphad assessment, perfectly represents the experimental carrier concentration at moderate and high temperatures. It shows that experimentally, for *T* > 500 K, the carrier concentration of NiTiSn can mainly be due to intrinsic Ni_i_ defects. In this case, since the defects change the DOS of NiTiSn by adding levels in the bandgap, it is important to choose the d-DOS rather than the p-DOS in order to perform these calculations. Moreover, the choice of the phase equilibria in the calculation of the energy of formation of charged defects has also an impact on the value of *N* even if it is minor. Finally, with the most representative combination: d-DOS and the Calphad phase diagram, we can provide an accurate description of *N* as a function of temperature. In the following sections, this combination will be chosen to estimate the associated thermoelectric properties.

### 5.2. Thermoelectric Properties

The Seebeck coefficient has been calculated by solving the Boltzmann’s equations using the BotzTraP code. For that, we have combined the previously calculated *N* with the band structure obtained from the cell with the defect (to be consistent with the method of determination of *N*). As stated in Section 3, the gap is slightly underestimated by the GGA for the Ni_i_ intrinsic defect. This can be corrected by a rigid band shift in the BoltzTraP code. We have adjusted the gap at 0.12 eV for all the temperatures. The obtained thermoelectric properties are compared with measurements [35,36,37,38] selected in our previous paper [4].

**Figure 5:**
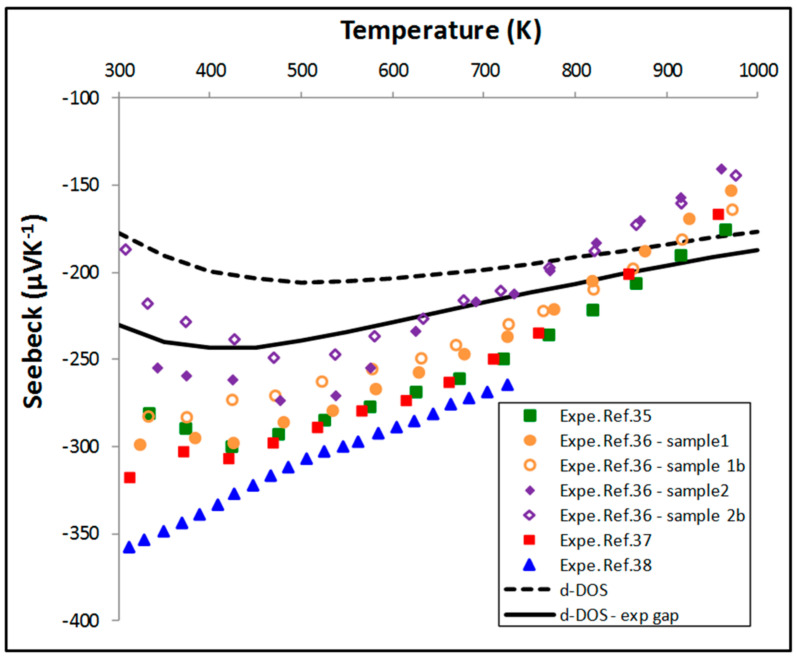
Evolution of the Seebeck coefficient calculated using BoltzTraP compared to a selection of experimental data.

With the calculated band gap (0.08 eV), if the Seebeck coefficient (Figure 5) has globally a correct shape as a function of temperature, the absolute values underestimate the measurements at low temperature. The agreement with measurements is improved by opening the gap in BoltzTrap to the experimental value of 0.12 eV. It can be noticed that even if the calculated carrier concentrations are similar to those measured by Muta et al. [35], even with the experimental gap it is not possible to perfectly reproduce the Seebeck values measured by Muta et al. [35]. The difference at low temperature may be partially due to an underestimation of the carrier concentration as shown in Figure 4b.

**Figure 6:**
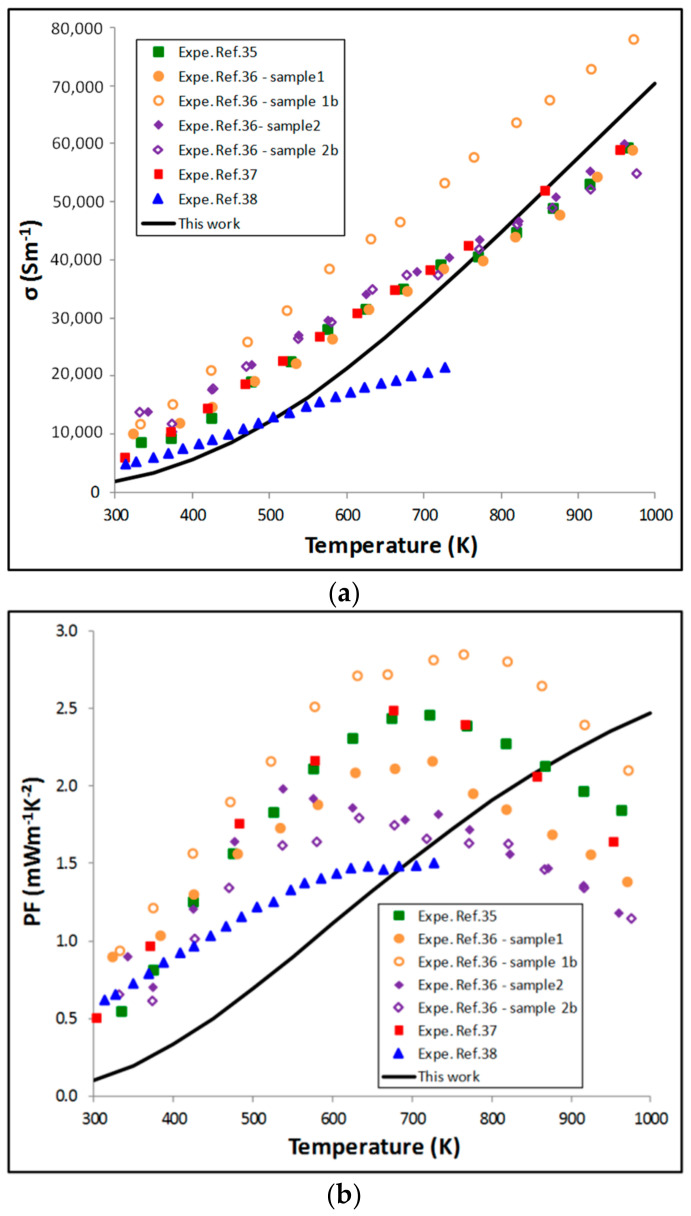
Effect of temperature on: (**a**) Electrical conductivity; (**b**) power factor (*PF*) calculated using BoltzTraP compared to a selection of experimental data.

Concerning the electrical conductivity, in BoltzTraP, the constant relaxation time approximation is taken into consideration and *σ/τ* (where *τ* is the electronic relaxation time) is calculated. In this study, we have assumed that *τ* does not change with temperature. *τ* has been set at 6 × 10^−15^ s. In these conditions, there is approximately a good representation of the shape of the evolution of *σ* as a function of *T* (Figure 6a).

From the calculated Seebeck coefficient and electrical conductivity, we have estimated the evolution of the Power Factor (*PF = S^2^σ*) as a function of temperature (Figure 6b). The shape is consistent with the expected one: the *PF* increases up to a maximum value (around 1300 K, not shown here) and then decreases. If the calculated maximum of the *PF* (2.8 mW·m^−1^·K^−2^) is in agreement with the experimental ones (1.5 to 3 mW·m^−1^·K^−2^ depending on the sample) the temperature of the maximum is shifted towards a higher temperature (600 K above the experimental values).

It is difficult to clearly define the origin of the difference between experiments and calculations. Several issues may be pointed out. At first, we can note that below 450 K, our calculations underestimate the carrier concentration. A higher value of *N* (for example −1 × 10^19^ holes·cm^−3^) will increase the calculated *S* (−264 μV·K^−1^) and slightly increase *σ* (2620 S·m^−1^) which will improve the *PF* (to 0.18 mW·m^−1^·K^−2^). The second unknown may come from the real experimental value of the gap. There has been only one measurement in the literature and a slight increase of the gap will increase the calculated Seebeck, especially at low temperature. We have also to keep in mind that in the present calculations, the evolution of the gap with temperature has not been considered. At high temperature, the gap should be smaller and as a consequence the calculated Seebeck should be smaller. This phenomenon may slightly change the evolution of *S* as a function of temperature and the overall shape of the curve may be more consistent with the measurements at high temperatures. The last difficulty comes from the constant *τ* approximation. An improvement of these four “weak points” will certainly improve the experiments/calculations agreement. In addition, another critical point is the presence of small amounts of secondary phases in the measured samples. Indeed, even if we have carefully selected the samples which in the literature seem to be mainly composed of NiTiSn, a small fraction of free Sn in NiTiSn alloys is practically always observed. This will also have an influence on the measured thermoelectric properties of NiTiSn. Taking into consideration all these limitations, we can state that with the present methodology, we are able to give a correct description of the Power Factor.

**Figure 7:**
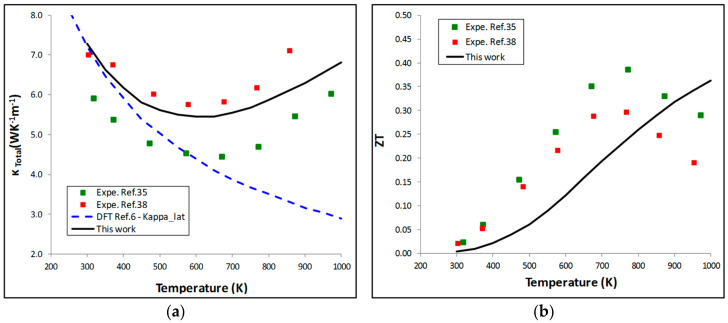
Evolution with temperature: (**a**) The thermal conductivity; (**b**) the figure of merit.

The BoltzTraP code also permits to calculate the electronic part of the thermal conductivity (*κ_e_*) divided by *τ*. Using the previous value of *τ*, *κ_e_* is estimated. This value is added to the lattice thermal conductivity (*κ_l_*) to obtain the total thermal conductivity *κ* (*κ = κ_e_ + κ_l_*). In this work, *κ_l_* has not been re-calculated and the previous calculations of Hermet et al. [6] have been taken into consideration. The thermal conductivity is highly impacted by grain boundaries and the grain size has to be taken into consideration in *ab initio* methods. In the work of Hermet et al. [6], a mean grain size of 175 nm allows to reproduce the experimental values of *κ_l_* at low temperature. These values (dashed lines) were added to our calculated *κ_e_* and the resulting global thermal conductivity (Figure 7a) shows a good agreement with measurements [35,38].

Finally, the thermal conductivity is combined to the *PF* to calculate the *ZT* of NiTiSn (Figure 7b). Similarly to the *PF*, the *ZT* curve has a correct shape with a maximum value around 0.42 in good agreement with measurements (0.3–0.4 [35,38]). However, similarly to the *PF* curve, the maximum of the *ZT* curve is calculated at 1300 K whereas experimentally the maximum values are measured around 775 K. This shift in the calculated *ZT* curve is mainly due to the shift of the maximum in the *PF* curve whose origin has already been discussed previously.

## 6. Conclusions

In this study, the electronic and thermoelectric properties of NiTiSn have been calculated via an *ab initio* method. From this study it appears that the main defects (interstitial Ni atoms) can be responsible of the electronic structure (band gap) and of the electronic properties of the pure material (in particular its n type semiconducting behavior). This crucial role of interstitial Ni atoms in NiTiSn half-Heusler compounds confirms recent experimental results [11,12,13]. In addition we have shown that a careful selection of the phase equilibria of the involved systems is necessary to provide a correct determination of the charge carrier concentration. Even if our fully *ab initio* procedure does not perfectly represent the thermoelectric properties of NiTiSn, it allows to give a correct description of the *PF* and of the *ZT* with very few experimental input data. The limits of the present method are mainly due to two approximations. Firstly the relaxation time *τ* has to be extrapolated from experimental measurements of *σ*. Secondly, the grain size of the samples has to be chosen to correctly reproduce the thermal conductivity. Moreover, we are aware that, experimentally, NiTiSn samples are often reported with secondary phases (Sn, Ni_2_TiSn…) due to synthesis issues. However our selection of data among the numerous experimental thermoelectric properties has been performed to limit this effect by rejecting the less pure samples. Finally in spite of these limitations, the present method gives a correct representation of the thermoelectric properties of NiTiSn and is thus well suited for predicting on the computer the effect of dopants (intrinsic or extrinsic) on the thermoelectric properties of a given material.

## Figures and Tables

**Table 1:** Calculated energy of formation of non-charged Ni_i_ defects in NiTiSn calculated within the GGA.

Phase Diagram	*Δ_def_E_s_* (eV)	*Δ_def_E_μ_* (eV) Calculated for Each 3-Phase Region (See Figure 2)									*Δ_def_E_multi_* (eV)
		1	2	3	4	5	6	7	8	9	
GGA	0.802	1.053	1.142	1.079	1.037	0.860	0.729	0.473	0.659	0.847	0.473
Calphad		1.172	1.036	0.984	0.405	0.405	0.753	-	-	-	0.405
